# Bidirectional Mendelian Randomization Analysis to Study the Relationship Between Human Skin Microbiota and Radiation-Induced Skin Toxicity

**DOI:** 10.3390/microorganisms13010194

**Published:** 2025-01-17

**Authors:** Hui Chen, Xiaojie Xia, Kexin Shi, Tianyi Xie, Xinchen Sun, Zhipeng Xu, Xiaolin Ge

**Affiliations:** 1 Department of Radiation Oncology, The First Clinical Medical College, Nanjing Medical University, Nanjing 210029, China; chenhui0307@163.com (H.C.); xiaxiaojie202@163.com (X.X.); kxshi106@163.com (K.S.); sunxinchen2012@163.com (X.S.); 2 Department of Neuroscience, Kenneth P. Dietrich School of Arts & Science, University of Pittsburgh, Pittsburgh, PA 15260, USA; xtytony2004@gmail.com; 3 Department of Urology, The First Clinical Medical College, Nanjing Medical University, Nanjing 210029, China

**Keywords:** radiation-induced skin toxicity, human skin microbiota, mendelian randomization, causal effect, GWAS

## Abstract

Radiation-induced skin toxicity, resulting from ionizing or nonionizing radiation, is a common skin disorder. However, the underlying relationship between skin microbiota and radiation-induced skin toxicity remains largely unexplored. Herein, we uncover the microbiota–skin interaction based on a genome-wide association study (GWAS) featuring 150 skin microbiota and three types of skin microenvironment. Summary datasets of human skin microbiota were extracted from the GWAS catalog database, and summary datasets of radiation-induced skin toxicity from the FinnGen biobank. Mendelian Randomization (MR) analysis was leveraged to sort out the causal link between skin microbiota and radiation-induced skin toxicity. We identified 33 causal connections between human skin microbiota and radiation-induced skin toxicity, including 19 positive and 14 negative causative directions. Among these potential associations, the genus *Staphylococcus* could serve as a common risk factor for radiation-induced skin toxicity, especially for radiodermatitis. And *Streptococcus salivarius* was identified as a potential protective factor against radiation-induced skin toxicity. Additional analysis indicated no pleiotropy, heterogeneity, or reverse causal relationship in the results. We comprehensively assessed potential associations of skin microbiota with radiation-induced skin toxicity and identified several suggestive links. Our results provide promising targets for the prevention and treatment of radiation-induced skin toxicity.

## 1. Introduction

Human skin is almost ubiquitously exposed to nonionizing radiation, such as ultraviolet (UV) light, radiofrequency waves, and microwaves [1,2]. Compared with the ionization effect of ionizing radiation, nonionizing radiation does not have sufficient energy, which may lead to thermal effects or non-thermal effects on human skin, such as skin photoaging [2,3]. Apart from nonionizing radiation, radiotherapy based on ionizing radiation is currently a pivotal treatment modality for most cancers, and it usually works as an independent clinical intervention or as a component in systematical therapy [4,5]. However, skin toxicity radiodermatitis at the irradiation site is one of the most common adverse effects, occurring in over 90% patients after radiotherapy and producing acute and chronic symptoms, including edema, erythema, pigmentation, hair loss, moist or dry desquamation, fibrosis, telangiectasia loss, and even skin cancer [6,7,8]. Radiodermatitis not only delays the healing process and increases the risk of infection or secondary tumor but also causes mental anxiety in patients by affecting the quality of life and decreasing their confidence in treatment [9,10]. Considering the non-negligible roles of radiation on human skin health, studying radiation-induced skin toxicity and its influencing factors is of significant importance to clinical prevention and treatment.

The diverse and complex microbial colonization of the human body has been proven to play a critical role in human health [11,12]. The skin, as the biggest organ of the human body, can not only resist microbial invasion but also acts as a home to its resident microbiota [13]. According to the various physiological features at different sites, human skin can be classified into dry, moist, and sebaceous microenvironments [14]. And the formation and maturation of the skin’s microbiota depend primarily on the different body sites and microenvironment [15]. Despite the dynamic interaction between human skin and the external environment, the symbiotic skin microbiota and the host maintain a peaceful and mutually beneficial relationship in a healthy state [16]. Meanwhile, previous studies have also validated that the pathogenesis of various skin diseases can result from or in skin microbial dysbiosis, including wounds, psoriasis, cutaneous lymphoma, atopic dermatitis, and so on [12,17,18].

With the intensive understanding of skin diseases, human skin microbiota have been considered as a novel therapeutic platform. For example, applying the autologous *Staphylococcus epidermidis* to facial skin was identified as an effective skin care method in a blinded randomized clinical trial (RCT) [19]. And some researchers have found that skin microbiome transplantation with probiotics can benefit in controlling odor [20]. As for radiation-induced skin toxicity, previous studies have almost completely focused on the mutual association between UV radiation and human skin microbiota and were constrained by confounding factors [21,22]. Therefore, in order to promote the clinical management of skin disorders caused by radiation, it is essential to clarify the mutual association between human skin microbiota and radiation-induced skin toxicity.

Mendelian randomization (MR), as a robust approach, takes genetic variants as instrumental variables and evaluates the potential causal effect of exposure on outcome [23]. Due to the randomized assignment of genetic variants to offspring at meiosis, MR can mitigate the bias rising from confounding factors, similar to a RCT study [24]. In our study, we performed a bidirectional two-sample MR to comprehensively and unprecedentedly estimate the causal links between human skin microbiota and radiation-induced skin toxicity on the basis of genome-wide association study (GWAS) data. Hopefully, our research can promote a comprehensive understanding of the potential factors influencing the pathogenesis of radiation-induced skin toxicity and provide valuable perspectives for future research and clinical management.

## 2. Materials and Methods

### 2.1. Data Sources for Human Skin Microbiota

Summary-level GWAS datasets were extracted from the meta-analysis research of a GWAS of European-ancestry populations from the EMBL-EBI GWAS Catalog database (https://www.ebi.ac.uk/gwas/, accessed on 8 June 2024, accession numbers from GCST90133164 to GCST90133313) (Table S1). The original meta-analysis research on a GWAS of human skin microbiota included 2 population-based German cohorts (KORA FF4 cohort and PopGen cohort) with European ancestry [25]. This meta-analysis research utilized the sequencing of V1-V2 regions from 16S rRNA gene to detect 1656 skin samples taken from three representative types of skin microenvironments (dry, moist, and sebaceous) and analyzed the association between the multivariate and univariate skin microbial features and variation in 4,685,714 human autosomal SNPs, which eventually generated the datasets about the relationship between different skin microbial features (consisting of 3 phyla, 4 classes, 7 order, 7 families, 15 genera, and 43 ASVs) and human host SNPs. According to different cohorts and skin sample sites, the summary GWAS statistics could be organized and divided into two series. Specifically, the skin samples included in series 1 were collected from the dorsal forearm (representing the dry skin microenvironment) in PopGen, antecubital fossa (representing the moist skin microenvironment) in KORA FF4, and forehead (representing the sebaceous skin microenvironment) in PopGen. And the skin samples included in series 2 were from the volar forearm (representing the dry skin microenvironment) in PopGen, antecubital fossa (representing the moist skin microenvironment) in PopGen, and retroauricular fold in KORA FF4.

### 2.2. Data Sources for Radiation-Induced Skin Toxicity

We included 3 series of summary GWAS data related to radiation-induced skin toxicity from the FinnGen database (https://r10.finngen.fi/, accessed on 18 December 2023; https://r7.finngen.fi/, accessed on 1 June 2022). The summary GWAS datasets were retrieved with 3 phenotypes including radiodermatitis (finngen_R10_L12_RADIODERMATITIS), radiation-related disorders of the skin and subcutaneous tissue (finngen_R7_L12_RADIATIONRELATEDSKIN), and skin changes due to chronic exposure to nonionizing radiation (finngen_R7_L12_NONIONRADISKIN). The details are shown in Table S2.

### 2.3. Screening of Instrumental Variables

**Figure 1:**
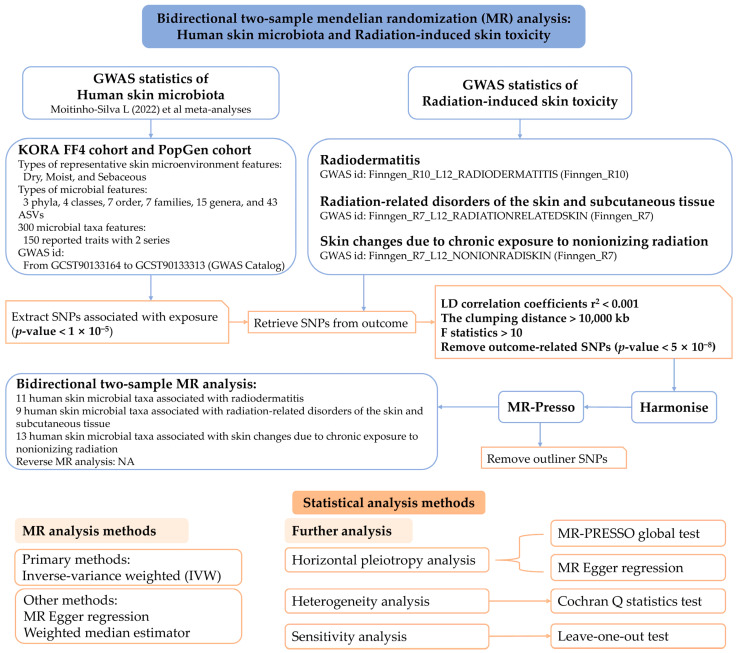
Flowchart of our MR analysis uncovering the causal associations between human skin microbiota and radiation-induced skin toxicity.

The workflow of bidirectional two-sample MR analysis in our study is illustrated in Figure 1. Initially, we selected the single nucleotide polymorphisms (SNPs) as instrumental variables, with a genome-wide statistical significance threshold of *p*-value < 5 × 10^−8^ from the GWAS datasets on human skin microbiota and radiation-induced skin toxicity. However, a very limited number of SNPs of human skin microbiota were filtered out via this threshold. In order to assess the causal relationships in a more comprehensive and wider range, we reselected the SNPs for human skin microbiota with a loose significance threshold of *p*-value < 1 × 10^−5^. The second set of SNPs for human skin microbiota was chosen for our MR analysis. A subsequent step to exclude the SNPs with linkage disequilibrium (LD) was conducted based on an LD correlation coefficient r^2^ < 0.001 and a clumping distance of 10,000 kb. In order to assess the bias from weak instrumental variables on casual inference, F-statistics of instrumental variables were calculated by using the formula F = R^2^/(1 − R^2^) × (N − K − 1)/K, and the instrumental variables with F-statistics < 10 were eliminated [26].

### 2.4. MR Analysis and Statistical Analysis

Our bidirectional two-sample MR analysis began with the harmonization of the SNPs with the same allele in the datasets [27]. Next, we performed the MR analysis via some common methods, including MR Egger regression [28], weighted median estimator [29], and inverse-variance weighted (IVW) method [30]. Among them, IVW was taken as the primary method with the highest efficiency [31]. The MR-PRESSO global test and MR Egger regression were applied to detect the potential horizontal pleiotropy, which existed if the *p*-value for the Egger-intercept < 0.05. The Cochran Q statistics test was used to evaluate the heterogeneity among instrumental SNPs. Further, the ‘leave-one-out’ test was performed to identify the potential outliners by excluding every instrumental SNP in turn. In order to evaluate the reverse causality, the reverse MR analysis was executed by taking radiation-induced skin toxicity as the exposure and human skin microbiota as the outcome. Our study was performed according to the Strengthening the Reporting of Observational Studies in Epidemiology Using Mendelian Randomization (STROBE-MR) checklist [32]. All the statistical analysis was accomplished and visualized on the basis of R (version 4.4.0), with the R packages ‘TwoSampleMR’, ‘ggplot2’, ‘foreach’, and so on.

## 3. Results

### 3.1. Screening of Instrumental Variables

With the locus-wide significance threshold (*p*-value < 1 × 10^−5^) and the LD threshold (r^2^ < 0.001, 10,000 kb from the index SNP), we screened out the SNPs as instrumental variables for human skin microbiota involving phyla, classes, orders, families, genera, and ASVs. As shown in the Table S3, the F-statistics for all the instrumental variables were greater than 10, suggesting that no weak index variant was included in our study.

### 3.2. Two-Sample MR Analysis of Human Skin Microbiota on Radiation-Induced Skin Toxicity

**Figure 2:**
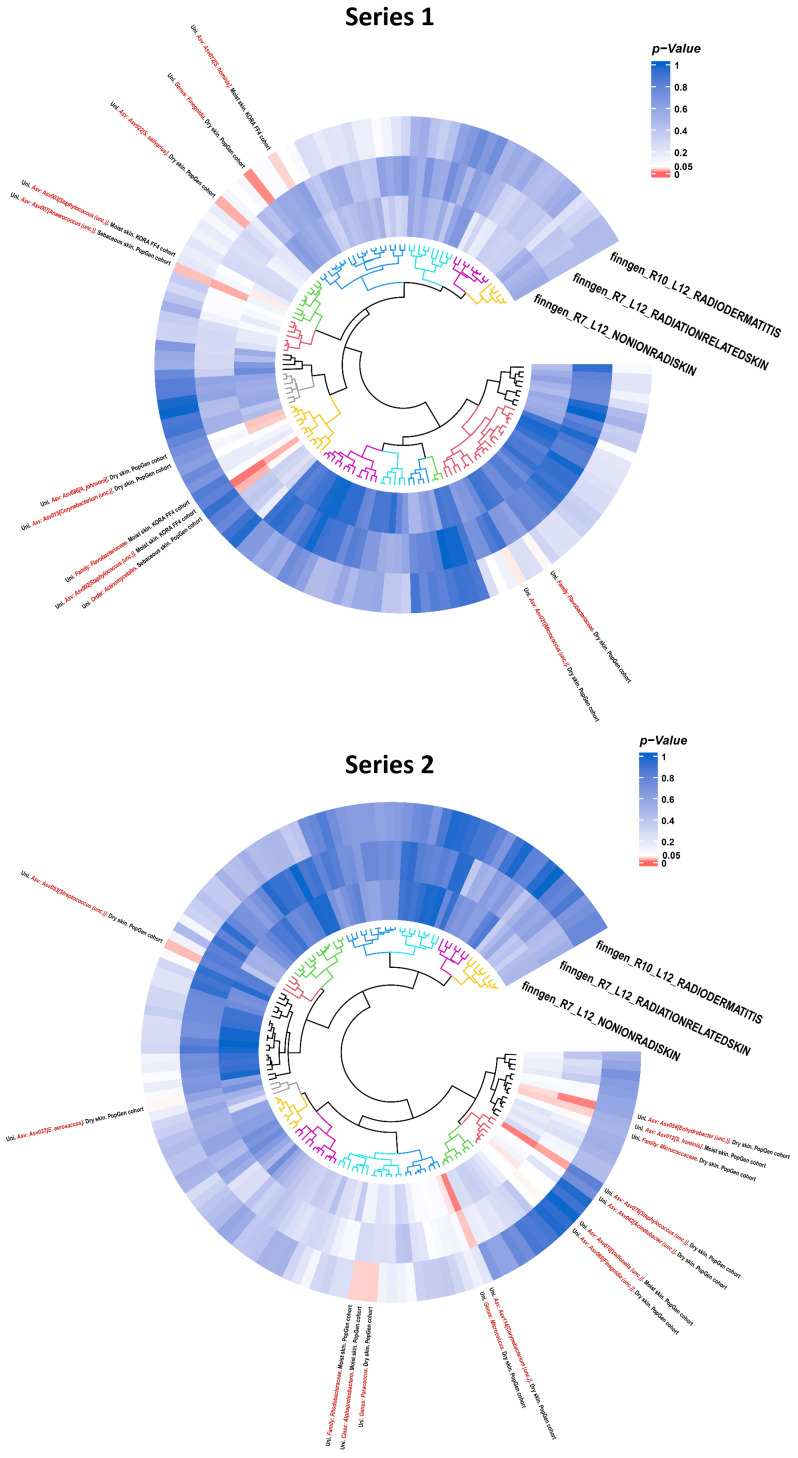
Overview of the suggestive causal effect of human skin microbiota (300 related GWAS datasets including 150 reported traits with 2 series) on radiation-induced skin toxicity (3 related phenotypes) detected by IVW method. Up, series 1; down, series 2. (Red color represents the statistical significance *p*-value < 0.05).

In our study, a total of 33 causal associations were determined via the bidirectional two-sample MR analysis between human skin microbiota (300 related GWAS datasets including 150 reported traits with two series) and radiation-induced skin toxicity (three related GWAS datasets). The overview of our results is displayed in Figure 2 and Table 1.

#### 3.2.1. Causal Impact of Human Skin Microbiota on Radiation-Related Disorders of the Skin and Subcutaneous Tissue

**Figure 3:**
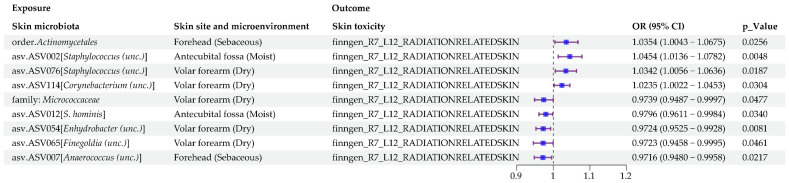
Significant MR estimates of the causal effects of human skin microbiota on radiation-related disorders of the skin and subcutaneous tissue.

**Figure 4:**
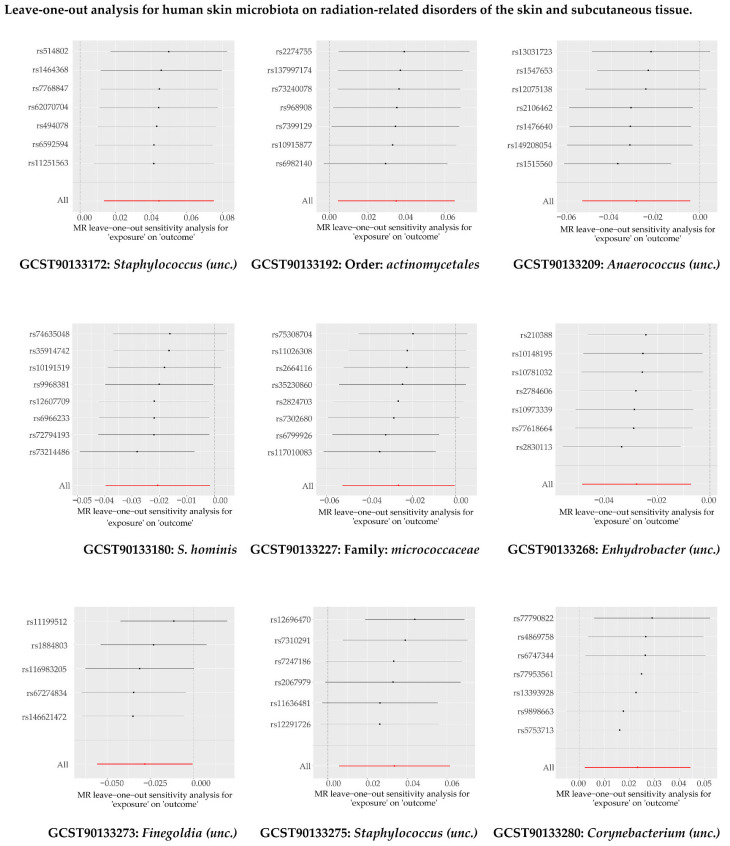
The ‘leave-one-out’ analysis for human skin microbiota on radiation-related disorders of the skin and subcutaneous tissue.

In our MR analysis, we found causal relationships between nine human skin microbial taxa (exposure) and radiation-related disorders of the skin and subcutaneous tissue (outcome) (Figure 3 and Table S4). Meanwhile, we observed no horizontal pleiotropy or heterogeneity (Table S5). The results of the sensitivity analysis are shown in Figure 4.

The IVW estimates revealed that four human skin microbial taxa were significantly associated with an increasing risk of radiation-related disorders of the skin and subcutaneous tissue. The details are as follows: order. *Actinomycetales* in sebaceous skin microenvironment (OR = 1.0354, 95% CI: 1.0043–1.0675, *p* = 2.5554 × 10^−2^); asv.ASV002 [*Staphylococcus (unc.)*] in moist skin microenvironment (OR = 1.0454, 95% CI: 1.0136–1.0782, *p* = 4.8275 × 10^−3^); asv.ASV076 [*Staphylococcus (unc.)*] in dry skin microenvironment (OR = 1.0342, 95% CI: 1.0056–1.0636, *p* = 1.8712 × 10^−2^); and asv.ASV114 [*Corynebacterium (unc.)*] in dry skin microenvironment (OR = 1.0235, 95% CI: 1.0022–1.0453, *p* = 3.0431 × 10^−2^).

On the other hand, five human skin microbial taxa showed a suggestively protective effect on radiation-related disorders of the skin and subcutaneous tissue. The details are as follows: family. *Micrococcaceae* in dry skin microenvironment (OR = 0.9739, 95% CI: 0.9487–0.9997, *p* = 4.7691 × 10^−2^); asv.ASV007 [*Anaerococcus (unc.)*] in sebaceous skin microenvironment (OR = 0.9716, 95% CI: 0.9480–0.9958, *p* = 2.1701 × 10^−2^); a.ASV012 [*S. hominis*] in moist skin microenvironment (OR = 0.9796, 95% CI: 0.9611–0.9984, *p* = 3.3997 × 10^−2^); asv.ASV054 [*Enhydrobacter (unc.)*] in dry skin microenvironment (OR = 0.9724, 95% CI: 0.9525–0.9928, *p* = 8.0967 × 10^−3^); and asv.ASV065 [*Finegoldia (unc.)*] in dry skin microenvironment (OR = 0.9723, 95% CI: 0.9458–0.9995, *p* = 4.6118 × 10^−2^).

#### 3.2.2. Causal Impact of Human Skin Microbiota on Skin Changes Due to Chronic Exposure to Nonionizing Radiation

**Figure 5:**
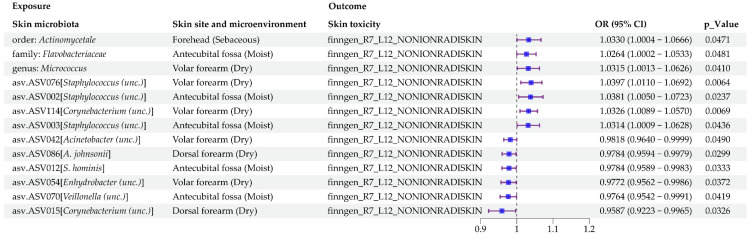
Significant MR estimates of the causal effects of human skin microbiota on skin changes due to chronic exposure to nonionizing radiation.

**Figure 6:**
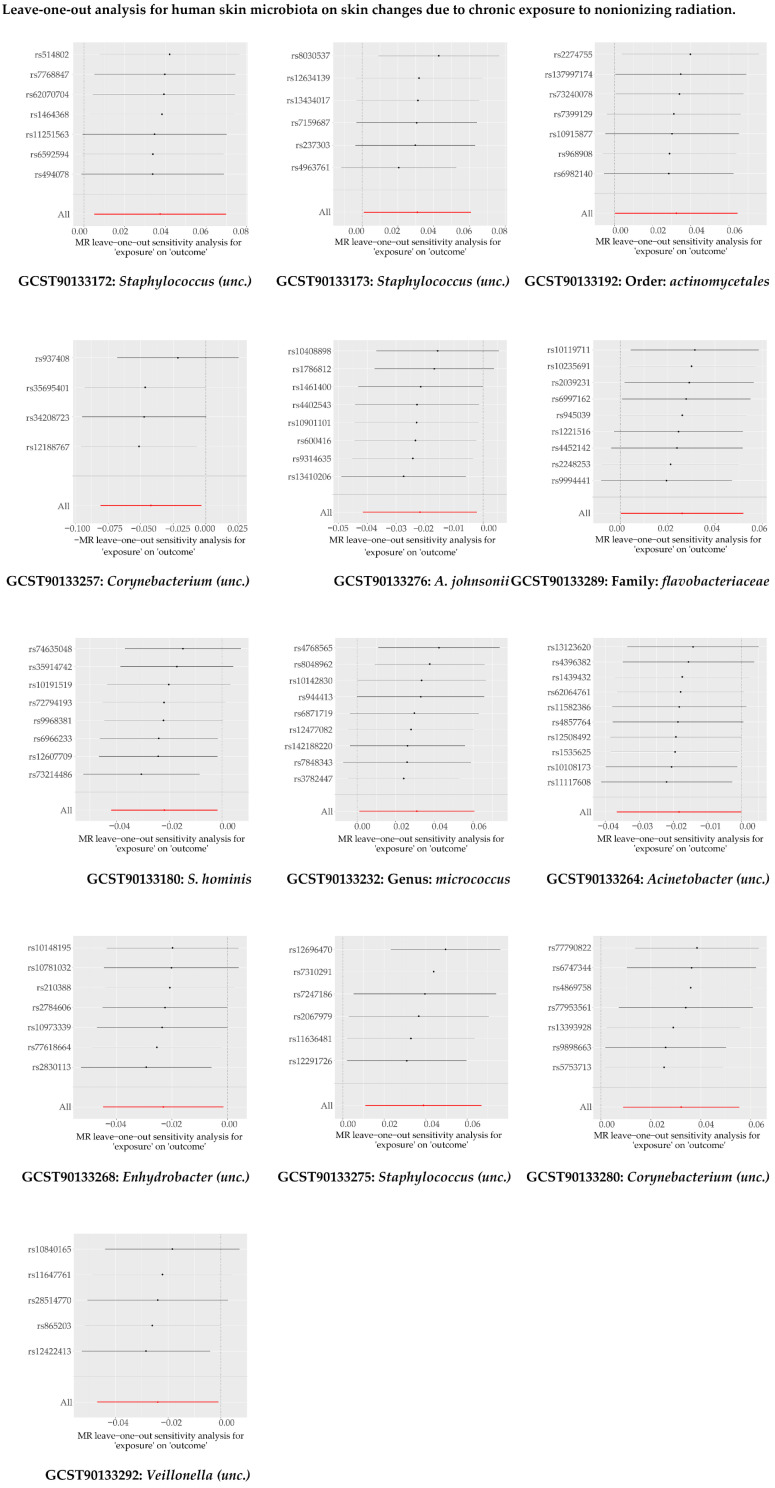
The ‘leave-one-out’ analysis for human skin microbiota on skin changes due to chronic exposure to nonionizing radiation.

Our two-sample MR analysis suggested 13 human skin microbial taxa (exposure) and skin changes due to chronic exposure to nonionizing radiation (outcome) (Figure 5 and Table S6). As shown in Table S5 and Figure 6, the robustness of our MR results was verified by horizontal pleiotropy, heterogeneity, and sensitivity tests.

The primary IVW method of MR analysis indicated that seven human skin microbial taxa were suggestively associated with an increased risk of skin changes due to chronic exposure to nonionizing radiation. The specific statistics are listed below: order. *Actinomycetales* in sebaceous skin microenvironment (OR = 1.0330, 95% CI: 1.0004–1.0666, *p* = 4.7147 × 10^−2^); family. *Flavobacteriaceae* in moist skin microenvironment (OR = 1.0264, 95% CI: 1.0002–1.0533, *p* = 4.8124 × 10^−2^); genus. *Micrococcus* in dry skin microenvironment (OR = 1.0315, 95% CI: 1.0013–1.0626, *p* = 4.1005 × 10^−2^); asv.ASV002 [*Staphylococcus (unc.)*] in moist skin microenvironment (OR = 1.0381, 95% CI: 1.0050–1.0723, *p* = 2.3715 × 10^−2^); asv.ASV003 [*Staphylococcus (unc.)*] in moist skin microenvironment (OR = 1.0314, 95% CI: 1.0009–1.0628, *p* = 4.3552 × 10^−2^); asv.ASV076 [*Staphylococcus (unc.)*] in dry skin microenvironment (OR = 1.0397, 95% CI: 1.0110–1.0692, *p* = 6.4051 × 10^−3^); and asv.ASV114 [*Corynebacterium (unc.)*] in dry skin microenvironment (OR = 1.0326, 95% CI: 1.0089–1.0570, *p* = 6.9147 × 10^−3^).

In addition, six human skin microbial taxa were suggestively associated with a lower risk of skin changes due to chronic exposure to nonionizing radiation. The specific statistics are as follows: asv.ASV015 [*Corynebacterium (unc.)*] in dry skin microenvironment (OR = 0.9587, 95% CI: 0.9223–0.9965, *p* = 3.2591 × 10^−2^); asv.ASV086 [*A. johnsonii*] in dry skin microenvironment (OR = 0.9784, 95% CI: 0.9594–0.9979, *p* = 2.9879 × 10^−2^); asv.ASV012 [*S. hominis*] in moist skin microenvironment (OR = 0.9784, 95% CI: 0.9589–0.9983, *p* = 3.3275 × 10^−2^); asv.ASV042 [*Acinetobacter (unc.)*] in dry skin microenvironment (OR = 0.9818, 95% CI: 0.9640–0.9999, *p* = 4.9003 × 10^−2^); asv.ASV054 [*Enhydrobacter (unc.)*] in dry skin microenvironment (OR = 0.9772, 95% CI: 0.9562–0.9986, *p* = 3.7176 × 10^−2^); and asv.ASV070 [*Veillonella (unc.)*] in moist skin microenvironment (OR = 0.9764, 95% CI: 0.9542–0.9991, *p* = 4.1942 × 10^−2^).

#### 3.2.3. Causal Impact of Human Skin Microbiota on Radiodermatitis

**Figure 7:**
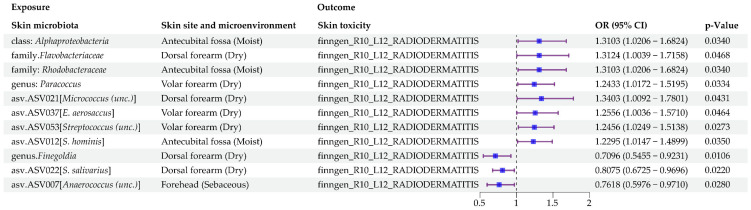
Significant MR estimates of the causal effects of human skin microbiota on radiodermatitis.

**Figure 8:**
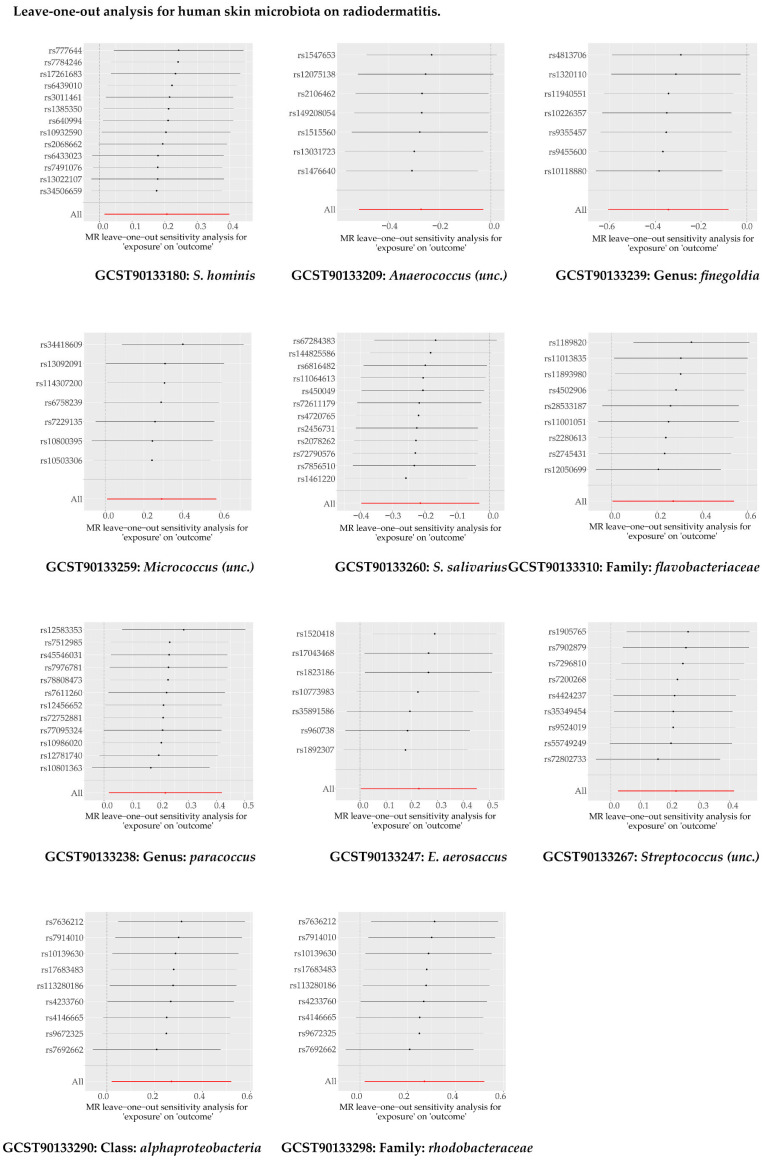
The ‘leave-one-out’ analysis for human skin microbiota on radiodermatitis.

As shown in Figure 7 and Table S7, 11 human skin microbial taxa (exposure) were identified as suggestive factors associated with the occurrence of radiodermatitis (outcome). No horizontal pleiotropy or heterogeneity were found in the results (Table S5). Further sensitivity analysis indicated no abnormality (Figure 8).

According to the primary MR estimate method, IVW analysis, eight human skin microbial taxa were significantly associated with an increased risk of radiodermatitis. The specific results are shown below: class. *Alphaproteobacteria* in moist skin microenvironment (OR = 1.3103, 95% CI: 1.0206–1.6824, *p* = 3.4046 × 10^−2^); family. *Flavobacteriaceae* in dry skin microenvironment (OR = 1.3124, 95% CI: 1.0039–1.7158, *p* = 4.6799 × 10^−2^); family. *Rhodobacteraceae* in moist skin microenvironment (OR = 1.3103, 95% CI: 1.0206–1.6824, *p* = 3.4046 × 10^−2^); genus. *Paracoccus* in dry skin microenvironment (OR = 1.2433, 95% CI: 1.0172–1.5195, *p* = 3.3441 × 10^−2^); asv.ASV012 [*S. hominis*] in moist skin microenvironment (OR = 1.2295, 95% CI: 1.0147–1.4899, *p* = 3.4968 × 10^−2^); asv.ASV021 [*Micrococcus (unc.)*] in dry skin microenvironment (OR = 1.3403, 95% CI: 1.0092–1.7801, *p* = 4.3059 × 10^−2^); asv.ASV037 [*E. aerosaccus*] in dry skin microenvironment (OR = 1.2556, 95% CI: 1.0036–1.5710, *p* = 4.6408 × 10^−2^); and asv.ASV053 [*Streptococcus (unc.)*] in dry skin microenvironment (OR = 1.2456, 95% CI: 1.0250–1.5138, *p* = 2.7266 × 10^−2^).

Meanwhile, three human skin microbial taxa were potentially associated with a decreased risk of radiodermatitis. The specific results are shown below: genus. *Finegoldia* in dry skin microenvironment (OR = 0.7096, 95% CI: 0.5455–0.9231, *p* = 1.0572 × 10^−2^); asv.ASV007 [*Anaerococcus (unc.)*] in sebaceous skin microenvironment (OR = 0.7618, 95% CI: 0.5976–0.9710, *p* = 2.7989 × 10^−2^); and asv.ASV022 [*S. salivarius*] in dry skin microenvironment (OR = 0.8075, 95% CI: 0.6725–0.9696, *p* = 2.1987 × 10^−2^).

### 3.3. Reverse Two-Sample MR Analysis

We further performed an evaluation of the reverse causation impact. In the reverse MR analysis, radiation-induced skin toxicity was used as the exposure, and human skin microbial taxa as the outcome. As shown in Table S8, radiation-induced skin toxicity exerted no causal impact on human skin microbial taxa, except a bidirectional causal relationship between skin changes due to chronic exposure to nonionizing radiation and asv.ASV002 [*Staphylococcus (unc.)*] in moist skin microenvironment (reverse MR: OR = 1.0381, 95% CI: 1.0050–1.0723, *p* = 2.3715 × 10^−2^).

## 4. Discussion

In recent years, interest regarding the inseparable correlation between the microbiome and human health has increased exponentially. For skin in particular, multiple studies have suggested that human skin microbiota contribute to the homeostasis of the skin microenvironment, the defense against pathogen invasion, and the education of the immune system [11,13]. To the best of our knowledge, our research is the first comprehensive evaluation of the causal association between human skin microbiota and radiation-induced skin toxicity. With the largest meta-analysis of GWAS on human skin microbiota conducted by Rühlemann MC et al. [25], we performed a two-sample MR analysis and identified 33 suggestive causal relationships between skin microbial taxa and radiation-induced skin toxicity. These human skin microbiota biomarkers may promote the understanding of radiation-related skin diseases and screening for potential therapeutic recommendations.

Based on bacterial 16S rRNA sequencing, previous researchers have demonstrated that the resident skin microbiota of healthy humans were composed mainly of the divisions *Proteobacteria*, *Firmicutes,* and *Bacteroidetes*, such as *Staphylococcus epidermidis* belonging to the *Firmicutes* division and *Pseudomonas* spp. belonging to the *Proteobacteria* division [33,34]. The diversity of human skin physical and chemical characteristics at different sites leads to the complex and dynamic homeostasis of skin microbial colonization [12]. However, the skin microorganisms with a peaceful, even mutually beneficial, relationship with humans can be pathogenic in specific settings, for instance, *Candida albicans*, *Staphylococcus epidermidis*, and *Staphylococcus aureus* infection in immunocompromised patients or skin lesions [35,36,37]. These opportunistic bacterial pathogens are unneglectable causes in most skin inflammatory diseases [13]. In our research, the genus *Staphylococcus* in the human skin microenvironment was demonstrated to be a common risk factor for all radiation-induced skin toxicity (three outcomes involved in our MR analysis), and especially for radiodermatitis (ASV012 [*S. hominis*] in moist skin microenvironment, OR = 1.230, 95% CI: 1.015–1.490, *p* = 3.497 × 10^−2^). Coincidentally, one clinical study with a small sample size conducted by Ramadan et al. also indicates that an overrepresentation of *Staphylococcus* is correlated with the chronicity and delayed healing of radiodermatitis [38]. Members of the genus *Staphylococcus*, one of the dominant bacteria present on human skin, are spherical Gram-positive bacteria and are related to both health and disease states [39,40]. Consistent with the GWAS data used in our study, *S. hominis* is one of the most abundant species of the genus *Staphylococcus* on human skin and it prefers moist skin sites [41]. In addition, just like other coagulase-negative *Staphylococcus* species, *S. hominis* has the features of biofilm formation and multi-drug resistance, which makes it a frequent opportunistic cause of local skin infection [42]. The potential pathogenic role of *S. hominis*, revealed by our MR analysis, provides a clue for mechanistic research on radiodermatitis. On the other hand, the complex interactions among various *staphylococcus* species have also attracted much attention in skin diseases. As reported, coagulase-negative *Staphylococcus* species can produce antimicrobial substances that inhibit other *staphylococcus* species and Gram-positive species in atopic dermatitis [43,44]. In psoriasis, a chronic skin inflammation, bacteria-derived extracellular vesicles can exert therapeutic effect by decreasing *Staphylococcus* colonization and further restoring the microbiota diversity on mice skin [45]. As for the treatment of skin diseases induced by radiation, the potential of targeting *Staphylococcus* species remains to be further investigated.

Apart from the genus *Staphylococcus* on human skin, most other risk factors in the pathogenesis of radiation-induced skin toxicity are also opportunistic pathogens. Specifically, the class *Alphaproteobacteria* and affiliated family *Rhodobacteraceae* exist in both healthy controls and patients, and the skin abundance of *Alphaproteobacteria* is associated with skin immunity and diseases [46,47,48,49]. It has been reported that the gut abundance of *Rhodobacteraceae* was positively related to the severity of chronic radiation enteritis in patients with cervical cancer after radiotherapy [50]. Combining our findings that the family *Rhodobacteraceae* existing on skin increases radiodermatitis risk (OR = 1.310, 95% CI: 1.021–1.682, *p* = 3.405 × 10^−2^), the intrinsic mechanism and role of *Rhodobacteraceae* in radiation-related diseases need to be further explored.

In addition, our MR analysis has identified the order *Actinomycetales*, family *Flavobacteriaceae*, genus *Paracoccus*, genus *Micrococcus*, ASV021 [*Micrococcus (unc.)*], ASV037 [*E. aerosaccus*] and ASV053 [*Streptococcus (unc.)*] as risk factors for radiation-induced skin toxicity. These bacteria are commonly found on human skin, mucosal membranes, and external environments and can act as opportunistic bacterial pathogens in many disease states, such as infection, autoimmune disease, and cancer [51,52,53,54,55]. Another dominant member of human skin microbiota, *Corynebacteria*, was associated with high risk of radiation-induced skin toxicity in our study. Belkaid et al. have uncovered that *Corynebacteria* on the skin can promote the abundance and activation of γδ T cells in the skin immune system, and this long-lasting effect of *Corynebacteria* is partially dependent on the immune factor IL-23 and independent of other microbes [56]. Notably, previous in vivo research has indicated that knockout of γδ T cells in mice can mitigate radiation-induced dermatitis [57]. We propose that γδ T cells in the skin or subcutaneous tissue may mediate the pro-inflammation role of *Corynebacteria* in the pathogenesis of radiation-induced skin toxicity. The above previous studies not only partially corroborate the reliability of our results in the present research but also suggest that the modulation of the skin immune system through targeting skin microbiota may be a potential direction for the prevention and treatment of radiation-related skin diseases, including radiodermatitis.

In our research, some skin bacteria are demonstrated as protective factors against radiation-induced skin toxicity. Corresponding to the findings on radiodermatitis in the present study (ASV022 [*S. salivarius*] in dry skin microenvironment, OR = 0.808, 95% CI: 0.673–0.970, *p* = 2.199 × 10^−2^), several studies have suggested that *Streptococcus salivarius* on the skin benefits skin immune homeostasis [58] and serves as a probiotic in psoriasis and atopic dermatitis [59,60]. *Streptococcus salivarius* can exert anti-inflammatory properties by inhibiting the NF-κB pathway and producing bacteriocins in the oral cavity and digestive tract, which suppress other pathogens and maintains immune homeostasis [61,62]. Further experimental research is necessary to validate the inhibitory role of *Streptococcus salivarius* on radiation-induced skin toxicity. A series of studies have revealed that *Veillonella* was abundant in healthy people cohort, and less colonization the gut or skin by *Veillonella* was detected in an atopic dermatitis or alopecia areata cohort compared to the healthy controls [63,64,65,66,67]. In our research, *Veillonella* was associated with a low risk of skin changes due to chronic exposure to nonionizing radiation. In addition, the anaerobic Gram-positive cocci *Anaerococcus* and *Finegoldia* are protective factors against radiodermatitis and radiation-related disorders of the skin and subcutaneous tissue, rather than skin changes due to chronic exposure to nonionizing radiation. Both *Anaerococcus* and *Finegoldia* are members of the family *Peptostreptococcaceae* and are common microflora on human skin in healthy and diseased states [51,68,69,70]. A negative causal effect of *Enhydrobacter* in the dry skin microenvironment against radiation-induced skin toxicity was detected in our MR analysis. However, the biological role of *Enhydrobacter* is controversial in skin disorders according to previous evidence, which has reported that the abundance of *Enhydrobacter* increases in seborrheic dermatitis lesions [71] and reduces in nail psoriasis and atopic dermatitis [72,73]. In recent years, researchers have been inspired by the beneficial effects of probiotics on microbiota–host homeostasis. Novel therapeutic approaches aimed at remodeling dysregulated microbiota and restoring compromised probiotics have been proven to be safe and effective in human dermatological diseases, such as acne vulgaris, diabetic infectious wounds, atopic dermatitis, psoriasis, and so on [74,75,76,77]. However, in the field of radiation-induced skin toxicity, researchers interested in host–microbiota interactions have focused almost exclusively on the interplay between human-associated microbiome and constant solar ultraviolet-induced injuries, which are usually attributed to the common type of nonionizing radiation [78,79,80]. The biological effect and underlying mechanisms of skin microbial communities in the pathogenesis of radiodermatitis resulting from the therapeutic ionizing radiation in clinical practice are yet to be systematically studied. Furthermore, although clinical studies have supported the safety and benefits of using topical prebiotics and postbiotics and oral probiotics for a variety of skin conditions, specific clinical guidelines do not currently exist [81]. Hence, designing clinical studies carefully to inform clinical practice is recommended. In addition, a previous study validated that cosmetic skincare products can interact with the skin microbiome and topical microbial balance [82], which could prompt researchers to carry out microbiological treatments by altering the host skin microenvironment.

The primary advantage of our research is that it is a pioneer in comprehensively elucidating the causal associations between human skin microbiota and radiation-induced skin toxicity via a bidirectional two-sample MR method. The MR investigation takes genetic variants as instruments in causal inference, and genetic variants are randomly distributed in populations due to the natural randomization of genetic variants during meiosis, which makes the MR study superior in alleviating potential reverse causation and residual confounders, similarly to the random distribution of samples seen in RCTs [24,27]. Secondly, the summary datasets on human skin microbiota utilized in our study included six hierarchical levels of microbial features from phyla to ASVs, which made it feasible to analyze the causal connections between skin microbiota and radiation-induced skin toxicity accurately and comprehensively. Thirdly, our findings were validated to be robust through a series of tests for horizontal pleiotropy, heterogeneity, and sensitivity. Overall, our research uncovers the role of skin microbiota on the pathogenesis of radiation-induced skin toxicity, which deepens our understanding of the importance of host–microbiome homeostasis in human health and diseases. Meanwhile, our findings provide clues for future exploration on the links between skin microbiota and radiation-induced skin toxicity and their underlying mechanisms. Therefore, further investigation is necessary to validate the potential of developing microbial therapies for radiation-induced skin toxicity, especially radiodermatitis, in clinical practice.

However, certain limitations in our study should be disclosed. Firstly, all of the GWAS summary statistics included in our study were of European ancestries, which may restrict the general applicability of our findings. Secondly, we found that some GWAS datasets utilized in our study at the ASV level of human skin microbiota pointed to the genera level, rather than the specific species level. Dedicated future GWAS research on skin microbiota is required to clarify the role of specific bacterial species. Thirdly, our research performed the MR analysis on the casual correlation between human skin microbiota and radiation-induced skin toxicity. In the MR analysis, instrumental variables were utilized to divide the population into subgroups, balancing confounding variables, analogous to the randomization step in RCTs. Hence, the MR method has been recognized as comparable to RCT studies in providing strong evidence to support causal relationships [83]. Nevertheless, before clinical translation of our findings, further experimental research based on in vivo and in vitro models is still necessary to clarify the functions of human skin microbiota and their interaction with hosts and environments.

## 5. Conclusions

**Figure 9:**
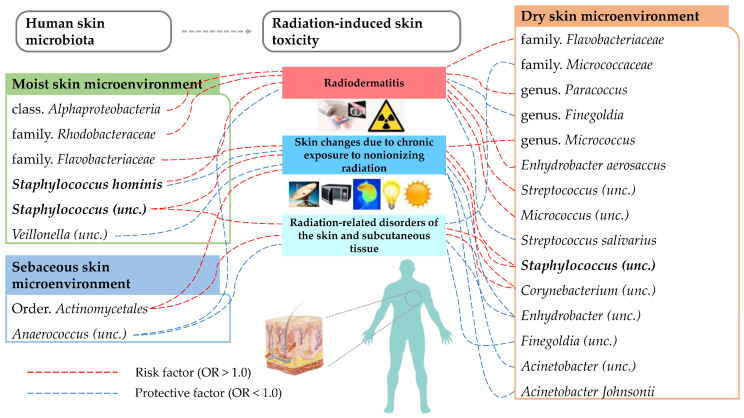
Our two-sample MR analysis revealed 33 suggestive causal relationships between skin microbial taxa and radiation-induced skin toxicity. Among them, the genus *Staphylococcus* in the human skin microenvironment was identified as a common risk factor for different types of radiation-induced skin toxicity. Red and blue lines represent the risk and protective factors for outcomes.

Our study comprehensively analyzed the causal relationships between human skin microbiota and radiation-induced skin toxicity, and we identified 33 suggestive causal relationships between skin microbial taxa and radiation-induced skin toxicity, involving 24 bacterial features (1 class, 1 order, 3 families, 3 genera, and 16 ASVs) (Figure 9). Our findings provide novel evidence about the role of human skin microbiota on radiation-induced skin toxicity, highlighting the potential of microbiota-based interventions in clinical practice around radiation-induced skin toxicity.

## Figures and Tables

**Table 1:** MR results of causal effects of human skin microbiota on radiation-induced skin toxicity.

ID.Exposure	Microbial Feature	Skin Microenvironment	ID.Outcome	*p*-Value	OR (95% CI)
GCST90133172	*Staphylococcus (unc.)*	Moist	Finngen_R7_L12_RADIATIONRELATEDSKIN	0.004828	1.0454 (1.0136–1.0782)
GCST90133192	Order: *actinomycetales*	Sebaceous	Finngen_R7_L12_RADIATIONRELATEDSKIN	0.025554	1.0354 (1.0043–1.0675)
GCST90133209	*Anaerococcus (unc.)*	Sebaceous	Finngen_R7_L12_RADIATIONRELATEDSKIN	0.021701	0.9716 (0.9480–0.9958)
GCST90133180	*S. hominis*	Moist	Finngen_R7_L12_RADIATIONRELATEDSKIN	0.033997	0.9796 (0.9611–0.9984)
GCST90133227	Family: *micrococcaceae*	Dry	Finngen_R7_L12_RADIATIONRELATEDSKIN	0.047691	0.9739 (0.9487–0.9997)
GCST90133268	*Enhydrobacter (unc.)*	Dry	Finngen_R7_L12_RADIATIONRELATEDSKIN	0.008097	0.9724 (0.9525–0.9928)
GCST90133273	*Finegoldia (unc.)*	Dry	Finngen_R7_L12_RADIATIONRELATEDSKIN	0.046118	0.9723 (0.9458–0.9995)
GCST90133275	*Staphylococcus (unc.)*	Dry	Finngen_R7_L12_RADIATIONRELATEDSKIN	0.018712	1.0342 (1.0056–1.0636)
GCST90133280	*Corynebacterium (unc.)*	Dry	Finngen_R7_L12_RADIATIONRELATEDSKIN	0.030431	1.0235 (1.0022–1.0453)
GCST90133172	*Staphylococcus (unc.)*	Moist	Finngen_R7_L12_NONIONRADISKIN	0.023715	1.0381 (1.0050–1.0723)
GCST90133173	*Staphylococcus (unc.)*	Moist	Finngen_R7_L12_NONIONRADISKIN	0.043552	1.0314 (1.0009–1.0628)
GCST90133192	Order: *actinomycetales*	Sebaceous	Finngen_R7_L12_NONIONRADISKIN	0.047147	1.0330 (1.0004–1.0666)
GCST90133257	*Corynebacterium (unc.)*	Dry	Finngen_R7_L12_NONIONRADISKIN	0.032591	0.9587 (0.9223–0.9965)
GCST90133276	*A. johnsonii*	Dry	Finngen_R7_L12_NONIONRADISKIN	0.029879	0.9784 (0.9594–0.9979)
GCST90133289	Family: *flavobacteriaceae*	Moist	Finngen_R7_L12_NONIONRADISKIN	0.048124	1.0264 (1.0002–1.0533)
GCST90133180	*S. hominis*	Moist	Finngen_R7_L12_NONIONRADISKIN	0.033275	0.9784 (0.9589–0.9983)
GCST90133232	Genus: *micrococcus*	Dry	Finngen_R7_L12_NONIONRADISKIN	0.041005	1.0315 (1.0013–1.0626)
GCST90133264	*Acinetobacter (unc.)*	Dry	Finngen_R7_L12_NONIONRADISKIN	0.049003	0.9818 (0.9640–0.9999)
GCST90133268	*Enhydrobacter (unc.)*	Dry	Finngen_R7_L12_NONIONRADISKIN	0.037176	0.9772 (0.9562–0.9986)
GCST90133275	*Staphylococcus (unc.)*	Dry	Finngen_R7_L12_NONIONRADISKIN	0.006405	1.0397 (1.0110–1.0692)
GCST90133280	*Corynebacterium (unc.)*	Dry	Finngen_R7_L12_NONIONRADISKIN	0.006915	1.0326 (1.0089–1.0570)
GCST90133292	*Veillonella (unc.)*	Moist	Finngen_R7_L12_NONIONRADISKIN	0.041941	0.9764 (0.9542–0.9991)
GCST90133180	*S. hominis*	Moist	Finngen_R10_L12_RADIODERMATITIS	0.034968	1.2295 (1.0147–1.4899)
GCST90133209	*Anaerococcus (unc.)*	Sebaceous	Finngen_R10_L12_RADIODERMATITIS	0.027989	0.7618 (0.5976–0.9710)
GCST90133239	Genus: *finegoldia*	Dry	Finngen_R10_L12_RADIODERMATITIS	0.010572	0.7096 (0.5455–0.9231)
GCST90133259	*Micrococcus (unc.)*	Dry	Finngen_R10_L12_RADIODERMATITIS	0.043059	1.3403 (1.0092–1.7801)
GCST90133260	*S. salivarius*	Dry	Finngen_R10_L12_RADIODERMATITIS	0.021987	0.8075 (0.6725–0.9696)
GCST90133310	Family: *flavobacteriaceae*	Dry	Finngen_R10_L12_RADIODERMATITIS	0.046799	1.3124 (1.0039–1.7158)
GCST90133238	Genus: *paracoccus*	Dry	Finngen_R10_L12_RADIODERMATITIS	0.033441	1.2433 (1.0172–1.5195)
GCST90133247	*E. aerosaccus*	Dry	Finngen_R10_L12_RADIODERMATITIS	0.046408	1.2556 (1.0036–1.5710)
GCST90133267	*Streptococcus (unc.)*	Dry	Finngen_R10_L12_RADIODERMATITIS	0.027266	1.2456 (1.0249–1.5138)
GCST90133290	Class: *alphaproteobacteria*	Moist	Finngen_R10_L12_RADIODERMATITIS	0.034046	1.3103 (1.0206–1.6824)
GCST90133298	Family: *rhodobacteraceae*	Moist	Finngen_R10_L12_RADIODERMATITIS	0.034046	1.3103 (1.0206–1.6824)

## Data Availability

The data supporting the findings of this study are publicly available from the EMBL-EBI GWAS Catalog database (accession numbers from GCST90133164 to GCST90133313) and FinnGen database (https://r10.finngen.fi/, accessed on 18 December 2023; https://r7.finngen.fi/, accessed on 1 June 2022) upon reasonable request. Data were processed using standard statistical methods implemented in R version 4.4.0. Details of data preprocessing, statistical analysis, and software tools are described in the Methods section of the manuscript. For additional inquiries, please contact the corresponding authors.

## Supplementary Materials

The following supporting information can be downloaded at https://www.mdpi.com/article/10.3390/microorganisms13010194/s1, Table S1: Summary of GWAS statistics for human skin microbiota from the EMBL-EBI GWAS Catalog database; Table S2: Summary of GWAS statistics for radiation-induced skin toxicity from FinnGen database; Table S3: Details of instrumental variables of human skin microbial features; Table S4: The causal effect of human skin microbiota on radiation-related disorders of the skin and subcutaneous tissue, evaluated by two-sample MR analysis; Table S5: The horizontal pleiotropy and heterogeneity tests of MR analysis between human skin microbiota (exposure) and radiation-induced skin toxicity (outcome); Table S6: The causal effect of human skin microbiota on skin changes due to chronic exposure to nonionizing radiation, evaluated by two-sample MR analysis; Table S7: The causal effect of human skin microbiota on radiodermatitis, evaluated by two-sample MR analysis; Table S8: The causal effect of radiation-induced skin toxicity on human skin microbiota, evaluated by reverse MR analysis.
